# ZnO Nanorods Based Enzymatic Biosensor for Selective Determination of Penicillin

**DOI:** 10.3390/bios1040153

**Published:** 2011-10-27

**Authors:** Zafar Hussain Ibupoto, Syed Muhammad Usman Ali, Kimleang Khun, Chan Oeurn Chey, Omer Nur, Magnus Willander

**Affiliations:** Department of Science and Technology, Campus Norrköping, Linköping University, Norrköping SE-60174, Sweden; E-Mails: syeal@itn.liu.se (S.M.U.A.); kimleang.khun@liu.se (K.K.); chan.oeurn.chey@liu.se (C.O.C.); omeno@itn.liu.se (O.N.); magnus.willander@itn.liu.se (M.W.)

**Keywords:** zinc oxide nanorods, enzyme penicillinase, potentiometric nanosensors, N-5-azido-2-nitrobenzoyloxysuccinimide, nanodevices

## Abstract

In this study, we have successfully demonstrated the fabrication of a biosensor based on well aligned single-crystal zinc oxide (ZnO) nanorods which were grown on gold coated glass substrate using a low temperature aqueous chemical growth (ACG) method. The ZnO nanorods were immobilized with penicillinase enzyme using the physical adsorption approach in combination with N-5-azido-2-nitrobenzoyloxysuccinimide (ANB-NOS) as cross linking molecules. The potentiometric response of the sensor configuration revealed good linearity over a large logarithmic concentration range from 100 µM to 100 mM. During the investigations, the proposed sensor showed a good stability with high sensitivity of ~121 mV/decade for sensing of penicillin. A quick electrochemical response of less than 5 s with a good selectivity, repeatability, reproducibility and a negligible response to common interferents such as Na^1+^, K^1+^, d-glucose, l-glucose, ascorbic acid, uric acid, urea, sucrose, lactose, glycine, penicilloic acid and cephalosporins, was observed.

## 1. Introduction

The determination and monitoring of antibiotics and other pharmaceutical compounds in the human body is of great interest to human health and to analytical and clinical chemistry, to veterinary, pharmaceutical and food industries. It is also very important during physiological and biochemical reactions occurring in the human body to know the penicillin concentration [1]. There are different techniques based on microbiological effects [2], various types of chromatographic [3,4], spectrophotometric [5,6], fluorimetric [7,8], and electro chemical approaches [9,10,11] to determine antibiotics such as Penicillin. A decisive critique about the detection of antibiotics in various samples is reported in [12]. Therefore, for measuring the metabolites in biomedical and biotechnological, biosensors are very practicable; for example in fermentation processes it is quite important to keep an eye on the concentration of penicillin [13]. The research carried out on enzyme field-effect transistors (EnFET) by Caras and Janata [14] has steered the trend towards potentiometric biosensors based on a semiconductor structure to become very popular [15,16,17]. Many potentiometric devices are based on various forms of FET devices to measure pH changes, selective ion concentrations, and the kinetics of biocatalytic reactions involving enzymes [18]. The conversion of a FET into a sensing device normally involves the replacement of the metal gate electrode by a biochemically sensitive surface (e.g., an analyte-selective membrane, an enzyme layer or an ion-conductive solution, *etc*.), which is brought into contact with the solution to be sensed [19]. A reference electrode is also present in the analyte solution, which completes the circuit via the gate voltage bias [20,21]. One of the most popular methods for the construction of FET-based biosensing devices is the immobilization of enzymes at the gate surface of pH-sensitive ion-sensitive FET (ISFET) devices. In the past, a biosensor based on the penicillinase enzyme immobilization on the surface of transducer by homo-functional cross-linking with glutaraldehyde method, has been reported [22]. Mostly, potentiometric biosensors were based on homo-functional cross-linking method for the immobilization for the penicillinase enzyme. However, such a method of immobilization has some disadvantages and the biosensors developed by this method have poor stability and quick stripping of enzyme molecules during the operation due to the weak adhesion of the membrane. The reason for this might be due to the inter enzyme molecules cross linking instead of the binding of the enzyme molecules to the transducer surface. There is also another method used for the immobilization of penicillinase enzyme which is the hetero-functional cross-linkers [23]. This is a two-step method, in the first step cross linker molecules bind to the pH sensitive transducer and after that enzyme molecules are added to them. The potentiometric technique is easy, quick, and of low cost for the detection of penicillin by using the ion selective electrodes. Moreover, potentiometric sensors have been used to detect individual as well as entire amounts in various β-lactam testing analytics [24,25]. ZnO is one of the most important semiconductor materials due to its many interesting properties. It is also important due to the availability of a variety of nanostructures such as nanowires, nanorods, nanotubes *etc*., which can be grown using the low temperature aqueous chemical growth (ACG) method [26,27]. There are numerous applications of ZnO nanorods in the fields of bioelectronics and nanoelectronics and they are frequently used for biosensing applications due to special properties such as high surface area to volume ratios, great chemical stability, biocompatibility, and that they are easy to grow and fabricate. Moreover, ZnO nanostructures are atoxic and provide fast electron communication [28,29,30,31,32,33,34]. ZnO nanomaterials can be used in a variety of electrochemical biosensing schemes due to their unique advantages in combination with immobilized enzymes. They can maintain the activity of the enzyme due to the desirable microenvironment, and enhance the direct electron transfer between the enzyme’s active sites and the electrode. The high isoelectric point (IEP) of ZnO (9.5) makes it a good matrix to immobilize low isoelectric point acidic proteins or DNA by electrostatic interactions with high binding stability [35,36,37]. In addition, ZnO has high ionic bonding (60%), and it dissolves very slowly at normal biological pH values. The enzyme penicillinase has a good electrostatic interaction with ZnO nanorods due to its low IEP at a pH of 6.9 and shows a good enzymatic activity and high selectivity to penicillin. 

In the present study, we have successfully demonstrated the fabrication of a ZnO nanorods based biosensor with good reproducibility and selectivity for quick monitoring of penicillin with immobilization of penicillinase enzyme by simple physical adsorption method.

## 2. Experimental Section

### 2.1. Materials

The penicillinase enzyme with given activity 1,500–3,000 U/mg-protein from Bacillus cereus, penicillin G sodium salt, Zinc nitrate hex hydrate Zn(NO_3_)_2_·6H_2_O and hexamethylenetetramine (HMT), sodium hydrogen phosphate (Na_2_HPO_4_), potassium dihydrogen phosphate (KH_2_PO_4_), sodium chloride (NaCl), potassium chloride (KCl), sodium hydroxide (NaOH) and hydrochloric acid (HCl) were purchased from (Sigma Aldrich). The N-5-azido-2-nitrobenzoyloxysuccinimide (ANB-NOS) cross linking chemical was purchased from (Pierce). All other chemicals were of analytical grade.

### 2.2. Preparation of Sensor Electrodes

**Figure 1:**
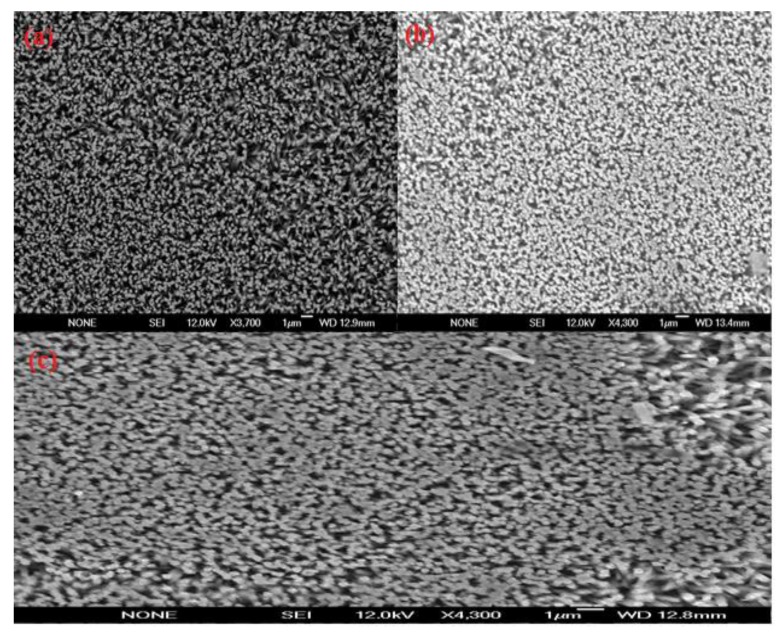
(**a–c**) Field emission scanning electron microscopy (FESEM) images of the ZnO nanorods grown on gold coated glass substrate using the aqueous chemical method (**a**) showing the as grown ZnO-nanorods, (**b**) with penicillinase immobilization, and (**c**) after use.

We have prepared the sensor electrodes on gold coated glassy substrates. First a borosilicate glass was cleaned and, then supplemented into vacuum chamber in an evaporator system (Satis CR 725). A thin film of 20 nm thickness of titanium was evaporated as an adhesive layer, and then 120 nm thickness of gold was evaporated. After these processes, well aligned and vertically oriented ZnO nanorods were grown on the gold coated glass by using a low temperature aqueous chemical growth method (ACG). For the growth process, the gold coated glass substrates were cleaned with acetone and deionize water and dried by nitrogen gas. Then a uniform layer of zinc acetate dihydrate seed solution was applied by spin coater at 2,500 rpm for 30 s. After that the substrates were annealed at 200 °C for 10 min. These annealed samples were affixed in sample holders and dipped in the 1:1 equimolar solution of zinc nitrate hexahydrate and hexamethylenetetramine (HMT) in a preheated oven maintained at 95 °C for 5–8 h. After the completion of the growth process the substrates were taken out from oven and cleaned with de-ionize water to remove the residual salt particles and dried with nitrogen gas. The morphology of the well aligned and vertically oriented grown ZnO nanorods was investigated by field emission scanning microscopy (FESEM ) as shown in Figure 1(a).

### 2.3. Immobilization of the Enzyme

Six sensor electrodes were prepared after the immobilization of enzyme penicillinase on to the surface of grown ZnO nanorods in combination with (ANB-NOS) as a cross linker. The process of immobilization followed two steps; first a 10 mM (ANB-NOS) solution was prepared in phosphate buffer at pH 7.4, then ZnO nanorods electrode was hatched in this solution for one hour, after that sensor electrode was washed with de-ionize water in order to remove the solid residue particles, then in second step, this electrode was put into the enzyme penicillinase solution for 20 min. The penicillinase enzyme solution was prepared in same phosphate buffer at pH 7.4 and concentration of enzyme was 5 mg/mL. The FESEM image of enzyme immobilized ZnO nanorods has shown in Figure 1(b). Then immobilized sensor electrode was kept at 4 °C temperature for about 16 hours. All immobilized sensor electrodes were kept at 4 °C temperature when not in use. The electrochemical measurements, using potentiometric method were carried out with an immobilized ZnO nanorods based sensor as working electrode and Ag/AgCl as a reference electrode by Metrohm pH meter (model 827).

## 3. Results and Discussion

The electrochemical cell voltage (EMF) *i.e*., the potential difference between the sensor electrode and the reference electrode (Ag/AgCl), changes with the variation in the composition of the penicillin test electrolyte solution. These changes in the resulting potential were attributed to the concentration of the penicillin in the test electrolyte solutions and the reaction of penicillinase enzyme. The electrochemical response of the penicillin biosensor depends on the extent of the catalytic activity of the penicillinase enzyme to penicillin. The hydrolysis reaction of penicillin G salt in presence of penicillinase enzyme is represented in the following equation:

Penicillin + H_2_O = penicilloate^−^ + H^1+^

**Figure 2:**
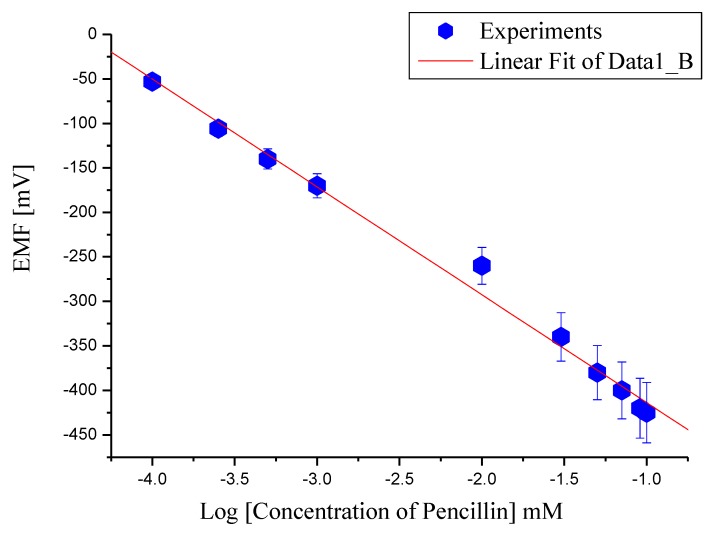
Calibration curve of the immobilized ZnO-nanorods based sensor electrode showing the electrochemical response (EMF) at various penicillin salt concentrations (100 µM to 100 mM) with Ag/AgCl reference electrode.

As a result of the above reaction, hydrogen ions (H^1+^) are produced and can be used to determine the penicillin concentration [38]. Due to the generation of (H^1+^) ions in the reaction, the pH of the solution also decreases. As the amount of charges produced around the ZnO nanorods based sensor electrode changes, a change into the electrode potential was observed [39]. The electrochemical measurements using the potentiometric method were carried out for different penicillin G salt concentrations varying from 100 µM to 100 mM made in a buffer solution at pH 7.4. The tested sensor configuration showed a wide linear dynamic range for the output response (EMF) *vs*. the logarithmic concentration of penicillin G salt solution as shown in Figure 2. We obtained a slope of 121 mV/decade, which is a manifestation of high specificity for the determination of penicillin.

### Performance Evaluation of the Sensor

**Figure 3:**
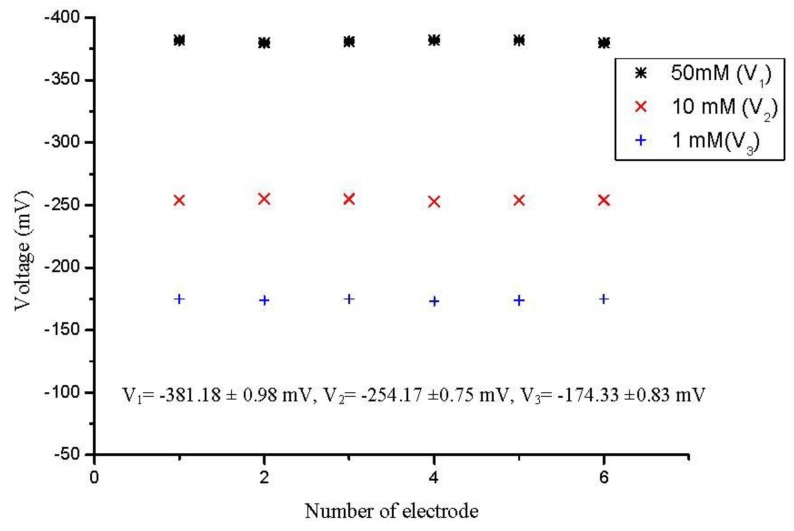
Showing the sensor to sensor reproducibility in 10 mM penicillin solution.

To assess the performance of a sensor it is important to evaluate different parameters such as possible concentration detection range, selectivity, detection limit, reproducibility, and response time *etc*. The reproducibility is a very important parameter for the performance evaluation of a sensor in order to know the consistency in working activity. During the present experiments, we fabricated six sensor electrodes independently using the same conditions and immobilized the enzyme onto the ZnO nanorods in order to study the reproducibility and life time stability of the proposed sensor. To investigate the reproducibility of the presented sensor, we checked the potentiometric response of all six sensor electrodes in 1, 10 and 50 mM penicillin solutions and the relative standard deviation was found to be less than 5% as shown in Figure 3.

**Figure 4:**
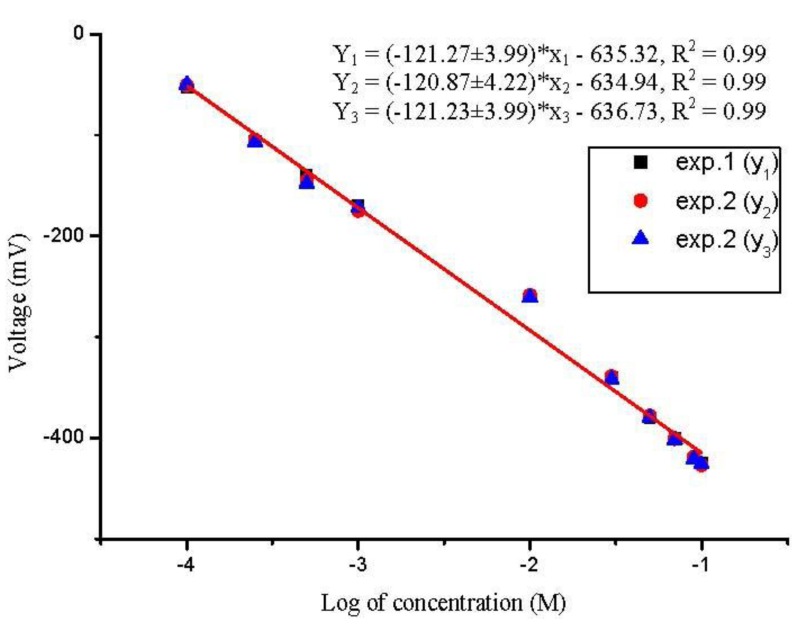
Calibration curves showing the biosensor repeatability at room temperature after 2–3 h passage of penicillin G salt solution from 100 µM to 100 mM concentration range.

In addition, the repeatability of the presented sensor was also evaluated by performing three experiments with the same sensor electrode for three consecutive days, and after each measurement the electrode was soaked in a phosphate buffer solution (PBS) and after that it was dried and stored at 4 °C. The sensor has revealed a good repeatability performance as shown in Figure 4.

**Figure 5:**
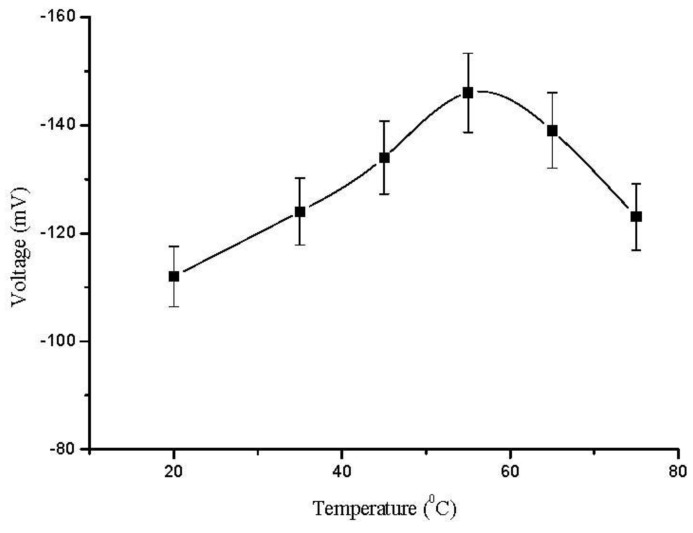
Calibration curves showing the investigation of EMF response with the change in temperature.

The morphology of the ZnO nanorods right after the measurements was checked with FESEM and it has been observed that the ZnO nanorods were intact with electrode surface. It was only observed that the ZnO nanorods were slightly dissolved from the tip due to minor change in the pH of the analyte test solution as shown in Figure 1(c). The effect of temperature on the performance of the sensor response was also studied by varying the temperature from 20 °C to 80 °C. The results are shown in Figure 5. During the experiments, a trend of gradual increase in the EMF response of sensor electrode with increasing temperature was observed, and it reached its maximum value at around 50 °C. This is due to the fact that the enzyme has its maximum activity at 50 °C and above 50 °C there was a sudden decrease in the EMF response of the sensor due to the heating effect on the immobilized enzyme, which degraded the enzyme functionality. However, the sensor showed maximum response at 50 °C but it was not as stable as at room temperature. Thus, we have chosen to work at room temperature 23 ± 2 °C for the ease of practical measurements and also to avoid evaporation of the solution.

**Figure 6:**
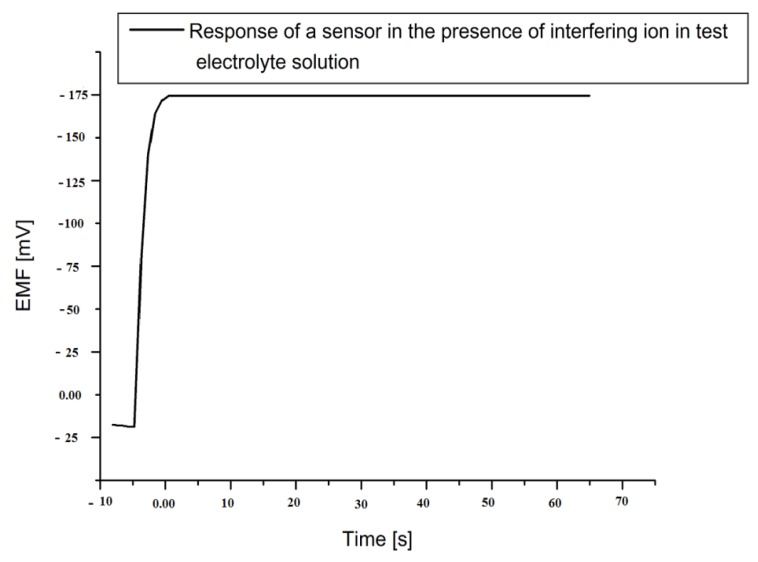
The time response curve of the proposed sensor in a 1,000 µM penicillin electrolytic test solution in presence of interfering species.

The selectivity is an important parameter for the performance evaluation of biosensors, as it gives the particular affinity for observing specific target ions in the presence of other interfering ions. The selectivity of any biosensor can be evaluated in an electrolyte test solution by adding other interfering ions. The enzyme penicillinase has quite acceptable selectivity [40]. For penicillin, the enzyme-analyte reaction is highly specific and produced the charged ions which were selectively determined by our proposed sensor. The penicillinase is very particular in reaction with penicillin, even in presence of other interfering species. We found no significant interference of sensor with Na^1+^, K^1+^, d-glucose, l-glucose, ascorbic acid, uric acid, urea, sucrose, lactose, glycine, penicilloic acid and cephalosporins. In Figure 6, it can be seen that upon mixing all above interfering components (100 µM of each) into 1 mM of our penicillin electrolyte solution, no changes on the output signal stability or magnitude was observed. Moreover, the sensor also exhibited a very fast response time of less than 5 s when the signal reached its steady state stable value. 

**Figure 7:**
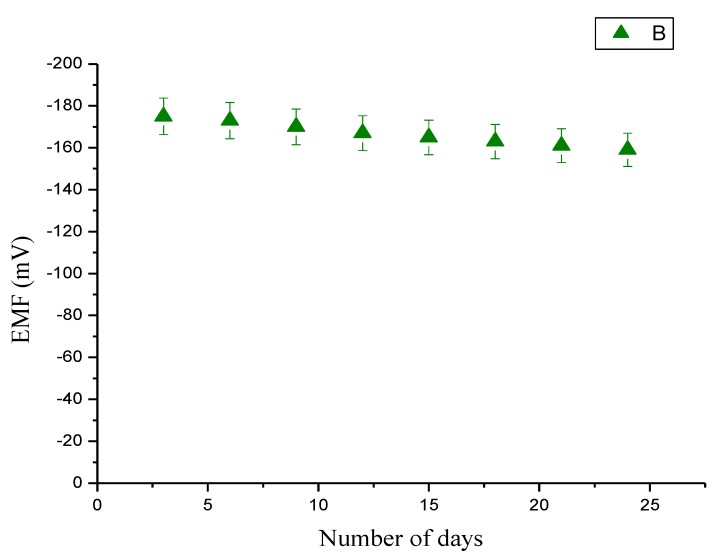
Calibration curve showing the study of the electromotive (EMF) response with the influence of storage at 4 °C for three weeks.

The storage stability of the presented sensor has been investigated with a series of experiments performed periodically for more than four weeks and the sensor electrodes were kept at 4 °C when not in use. It has been observed that the sensor retained their enzymatic activity up to 90% of their initial activities exhibiting good storage ability and reusability for a long period of time as shown in Figure 7.

## 4. Conclusions

We have successfully demonstrated the fabrication of a penicillin biosensor based on ZnO nanorods immobilized by penicillinase enzyme with a simple physical adsorption method in conjunction with (ANB-NOS) as a cross linking molecule for penicillinase enzyme. The proposed sensor exhibited good selectivity, reproducibility, sensitivity over a wide range of linear dynamic logarithmic concentrations (ranging from 100 µM to 100 mM) of the penicillin salt electrolyte solution. A good sensitivity of 121 mV/decade slope with a fast response time of ~5 s at room temperature was observed. Moreover, the proposed sensor retained its enzymatic activities for a long period of time due to the strong electrostatic interaction between the ZnO nanorods and the penicillinase enzyme. We believe that all these advantageous features will make this proposed sensor applicable in the fermentation, medical as well as for other related areas.
